# Investigations on Cellular Uptake Mechanisms and Immunogenicity Profile of Novel Bio-Hybrid Nanovesicles

**DOI:** 10.3390/pharmaceutics14081738

**Published:** 2022-08-20

**Authors:** Yi-Hsuan Ou, Jeremy Liang, Wei Heng Chng, Ram Pravin Kumar Muthuramalingam, Zi Xiu Ng, Choon Keong Lee, Yub Raj Neupane, Jia Ning Nicolette Yau, Sitong Zhang, Charles Kang Liang Lou, Chenyuan Huang, Jiong-Wei Wang, Giorgia Pastorin

**Affiliations:** 1 Department of Pharmacy, National University of Singapore, Singapore 117543, Singapore; 2 Department of Chemistry, National University of Singapore, Singapore 117543, Singapore; 3 Integrative Sciences and Engineering Programme, NUS Graduate School, National University of Singapore, Singapore 119077, Singapore; 4 Department of Biological Sciences, National University of Singapore, Singapore 117558, Singapore; 5 Department of Pharmaceutical Sciences and Experimental Therapeutics, College of Pharmacy, The University of Iowa, Iowa City, IA 52242, USA; 6 Department of Surgery, Yong Loo Lin School of Medicine, National University of Singapore, Singapore 119228, Singapore; 7 Nanomedicine Translational Research Programme, Centre for Nanomedicine, Yong Loo Lin School of Medicine, National University of Singapore, Singapore 117609, Singapore; 8 Cardiovascular Research Institute, National University Heart Centre, Singapore 117599, Singapore; 9 Department of Physiology, Yong Loo Lin School of Medicine, National University of Singapore, Singapore 117593, Singapore

**Keywords:** cellular uptake, endocytosis, immunogenicity, pro-inflammatory cytokines, bio-hybrids

## Abstract

In drug delivery, the development of nanovesicles that combine both synthetic and cellular components provides added biocompatibility and targeting specificity in comparison to conventional synthetic carriers such as liposomes. Produced through the fusion of U937 monocytes’ membranes and synthetic lipids, our nano-cell vesicle technology systems (nCVTs) showed promising results as targeted cancer treatment. However, no investigation has been conducted yet on the immunogenic profile and the uptake mechanisms of nCVTs. Hence, this study was aimed at exploring the potential cytotoxicity and immune cells’ activation by nCVTs, as well as the routes through which cells internalize these biohybrid systems. The endocytic pathways were selectively inhibited to establish if the presence of cellular components in nCVTs affected the internalization route in comparison to both liposomes (made up of synthetic lipids only) and nano-cellular membranes (made up of biological material only). As a result, nCVTs showed an 8-to-40-fold higher cellular internalization than liposomes within the first hour, mainly through receptor-mediated processes (i.e., clathrin- and caveolae-mediated endocytosis), and low immunostimulatory potential (as indicated by the level of IL-1α, IL-6, and TNF-α cytokines) both in vitro and in vivo. These data confirmed that nCVTs preserved surface cues from their parent U937 cells and can be rationally engineered to incorporate ligands that enhance the selective uptake and delivery toward target cells and tissues.

## 1. Introduction

In recent years, cell-based therapy has gained popularity as a promising approach for various diseases [1,2]; however, carcinogenic risk and immunogenicity are still major concerns when using any cell-based product as a therapeutic agent. Particular concerns have been surrounding the use of therapeutic cells (i.e., stem cells and immune cells), as cells can actively proliferate, and this unregulated cell division and growth can cause tumor formation [3]. Moreover, depending on the cell types, the unregulated paracrine signaling may also participate in unwanted side effects [4], such as activation of the immune system and/or tumor development. For instance, infusion of chimeric antigen receptor T (CAR T) cells may lead to cytokine release syndrome (CRS) [5], leading to the rapid elevation of various pro-inflammatory cytokines. CRS can be characterized by fever in mild cases and multiple organ failure in more severe ones [5,6]. Moreover, therapeutic cells (such as stem cells) can also stimulate existing tumor development: by modulating the immune system [7], these cells may provide a permissive environment for tumor growth, or they can even participate as supportive stromal cells by secreting growth factors [3,8].

Cell-derived products (such as extracellular vesicles or other therapeutic cell-derived systems) are generally considered to be a safer alternative than the direct introduction of cells in the body [9]. Even though these cell-derived systems do not involve viable cells as the final product, the use of cells during bioprocessing may still result in the presence of residual host cellular and nuclear materials, which can also pose immunogenic and oncogenic risks, especially when translated into clinical applications [10,11]. Furthermore, similar to any foreign substance, upon intravenous administration, these cell-derived components (particularly from an allogeneic source) are confronted with the immune system of the host: their interaction with immune cells such as macrophages may, in turn, provoke undesirable immune activation [12].

In this manuscript, we propose the development of a novel hybrid of cell-derived and liposomal systems, termed nano-Cell Vesicle Technologies (nCVTs), as a promising drug delivery platform for cancer therapy. Designed and constructed based on the underlying principles of synthetic liposomes (i.e., nanovesicles uniquely composed of lipids, and associated with complement activation and immunogenicity) [13] and cell-based drug delivery, this unique system has been shown to be capable of circumventing the flaws of both its predecessors with improved activity [14]. Indeed, nCVTs are expected to harness the benefits from the liposomal systems, such as having a robust and relatively easy production method, high loading capacity, and a tunable functionality; at the same time, they should preserve the advantages of a cell-derived drug delivery system (DDS), including having low immunogenicity, high biocompatibility, intrinsic targeting properties, and the ability to evade premature clearance by the immune system.

It is known that the cell membrane preserves some intrinsic targeting moieties from the parent cells; thus, using emptied cells in the form of cell ghosts (CGs) for production, instead of whole cells, may help to minimize the presence of other intracellular material and enhance the overall safety profile of nCVTs as drug carriers [15]. Besides ensuring safety, the CG approach is also an important step to improve the consistency in the downstream processing (i.e., extrusion for downsizing or other drug-loading procedures) [16]. Since the presence of the cellular contents such as cytoplasmic proteins may result in greater variation and higher tendency of aggregation of vesicles (which, in turn, causes precipitation in the downstream processes) [17], removing these interfering materials may help to ensure consistency and efficiency of the production. Although we used immortalized monocytic U937 cells as a prototype for nCVTs production in this work, similar protocols could be easily applied to other cell types [18]. Nonetheless, being a relatively new DDS, no reported study has yet been conducted with respect to the fate or the immunogenic profile of nCVTs. Herein, in this work, we investigated the cytotoxicity and the uptake mechanisms of nCVTs in several cell lines. We also evaluated the in vitro immunostimulatory potential of nCVTs by using mouse macrophages and the potential mechanisms of internalization by utilizing endocytic inhibitors. We found that nCVTs exhibited low immunogenicity in target cells, despite exhibiting a greater cellular uptake than their synthetic counterparts (liposomes) in various cell lines. These data were confirmed by a small in vivo experiment, whereby the injection of nCVTs did not alter the immune cell populations and did not stimulate the release of inflammatory cytokines in the animals’ blood. This corroborates the fact that key monocytic membrane proteins are retained on their surfaces, and this bestows nCVTs with intrinsic targeting abilities and greater uptake, while also preserving their biocompatibility. The elucidation of the uptake mechanisms, which mainly occurred through receptor-mediated endocytosis, will be the key to further enhancing the nCVTs’ properties. For example, specific membrane proteins associated with major internalization pathways could be overexpressed in selected cells prior to nCVTs formation in order to enhance the selectivity of nCVTs’ uptake in target cells, thus paving the way for the development of the next generation of drug delivery systems.

## 2. Materials and Methods

### 2.1. Material

Hyclone^TM^ trypan blue stain and BCA protein assay were purchased from ThermoFisher Scientific and used as purchase. A ThermoScientific microcentrifuge was used in the process of CG production from U937 monocytes. Cyanine 3 (Cy3) NHS monoester dyes were purchased from Kerafast and used as per manufacturer’s recommendations. Cholesterol and 1,2-dioleoyl-sn-glycero-3-phosphocholine (DOPC) were purchased from Avanti Polar Lipids. A BioTek Synergy H^1^ Hybrid microplate reader was used for fluorescence measurements. Extrusion was performed with a Genizer Jacketed Gextruder (10 mL).

DOPC and cholesterol used in the production of nCVTs and liposomes were obtained from Avanti Polar Lipids (Alabaster, AL, USA). Filter membranes used during extrusion were purchased from GE Healthcare Life Sciences (Marlborough, MA, USA). DNA isolation was performed by using the DNeasy Blood & Tissue Kits from Qiagen (Hilden, Germany), following the manufacturer’s protocol. Measure-IT^TM^ High-Sensitivity Nitrite Assay Kit was purchased from ThermoFisher Scientific (Waltham, MA, USA). ELISA kits for measuring interleukin-6 (IL-6) and Tumor Necrosis Factor-α (TNF-α) were obtained from Biolegend (ELISAmax™, San Diego, CA, USA).

### 2.2. Cell Culture

U937 cells, HEK293 cells, and HeLa cells were a kind gift from Associate Professors Gigi Chiu and Wee Han Ang (NUS). RAW264.7 (mouse macrophages) and CT26 cells were obtained from ATCC. RAW264.7, CT26, and U937 cells were cultured in RPMI-1640 supplemented with 10% FBS. HEK293 was grown in DMEM supplemented with 10% FBS. All cells were maintained in a 5% CO_2_ incubator at 37 °C.

### 2.3. “Cell Ghost” (CG) Production

The production of nCVT involves the fusion of cellular components of monocytes, such as membrane-bound proteins, with synthetic lipids. For the production of nCVTs, a cell “emptying” procedure was adopted to remove the intracellular components (i.e., cytoplasmic proteins and nuclear materials), while preserving the cell membrane in the form of cell ghosts (CGs). CG production method was adopted from our earlier work [17]. Briefly, U937 cells were harvested at 70% confluency and resuspended in a hypotonic solution (PBS/sucrose) that consisted of 0.25× phosphate buffer saline (PBS, pH 7.4) and 0.06% *w*/*v* sucrose. The cell dispersion was incubated at room temperature for 24 h and subsequently resuspended and incubated in and 0.06% *w*/*v* sucrose in 1× PBS solution. The suspension was then centrifuged at 3000× *g* and resuspended in 60% *w*/*v* sucrose in 1× PBS to yield CGs, which were kept at 4 °C until further use.

The number of CGs was determined by a hemocytometer with trypan blue staining. A protease inhibitor cocktail was added throughout the experiment according to the manufacturer’s recommendation (200× dilution).

### 2.4. Production of nCVTs, Liposomes, and Nano-CGs

A total of 2 mg of DOPC and cholesterol (70:30 mol%) was weighed and dissolved in chloroform, and a thin film was formed by using rotary evaporation. To label the vesicles, Cy3 or Cy5.5 (0.1 mol%) was added during the thin-film production. For the production of nCVTs, the 1 × 10^7^ of CGs were first resuspended in PBS, before being extruded with a 5 μm polycarbonate membrane filter. The extruded CGs were then used to rehydrate the lipid film. The mixture was sonicated at 37 kHz for at least 30 min. The reason for these parameters is that a sonication beyond 45 min was associated with the fragmentation of some CGs into smaller particles prior to the formation of nCVTs, affecting the reproducibility of nCVTs. Then the dispersion was extruded (jacketed extruder, Genizer^TM^) at 35 °C through a series of filters with the following diameter: 0.4, 0.2, and 0.1 μm.

Liposomes were produced in a similar way, but without the addition of CGs. Nano-CGs were produced by first labeling CGs with Cy3 or Cy5.5 dye prior to extrusion. All formulations were normalized to the same fluorescence level (Cy3 or Cy5.5) before use.

### 2.5. Liposomes and nCVTs Characterization

The size and zeta potential of nCVTs, liposomes, and nano-CGs were determined via dynamic light scattering, using a Malvern Zetasizer Nano series. Protein quantification was assayed by using a standard BCA protein kit. Cy3 normalization was performed via fluorescence quantification to a fixed fluorescence value for both nano-DDSs with a microplate reader at 540/570 nm.

### 2.6. Cell Viability

The cytotoxic effects of empty nCVTs, liposomes (LIPOs), and nano-CGs were evaluated by using the standard MTT assay. HeLa, CT26, RAW264.7, and HEK293 cells were seeded in 96-well culture plates at the density of 1 × 10^4^ cells/well and incubated overnight. The vesicles were normalized to the same number and lipid content. The cells were incubated with the nanovesicles for a period of 24–72 h at culture condition before adding the MTT reagent (0.5 mg/mL) diluted in serum-free medium. After 1 h of incubation with MTT, the medium was aspirated, and 100 μL/well of DMSO was added. Absorbance was measured by spectrophotometer at 570 nm. Cell viability was assessed and compared to the negative control (i.e., cells treated with PBS).

### 2.7. Nitric Oxide (NO) Production

ThermoFisher Measure-IT^TM^ High-Sensitivity Nitrite Assay Kit was utilized for the quantification of nitrites produced by RAW264.7 macrophages. RAW264.7 cells were seeded into transparent 96-well plates (1 × 10^4^ cells/well) and incubated overnight. RAW264.7 cells were treated with the respective amount of nCVTs and liposomes. Positive controls were also established by treating RAW264.7 cells with 10 or 100 ng/mL of lipopolysaccharides (LPSs). After the designated duration (24 h/72 h incubation), 10 µL of media from treated wells were aliquoted for the detection of NO as nitrates via a nitrite assay kit.

### 2.8. IL-6 and TNF-α Production

RAW264.7 cells (2.5 × 10^5^ cells/well) were seeded in a 6-well plate overnight prior to respective treatments for 72 h. Cell culture media were collected for cytokine quantification. Concentrations of IL-6 and TNF-α were measured by using ELISA (ELISAmax™ kit; Biolegend) according to the manufacturer’s protocols. The number of cytokines was calculated from standards provided by the manufacturer. Appropriate dilutions of samples were performed as per the manufacturer’s recommendations.

### 2.9. In Vivo Immunogenicity Profiling

All animal studies were approved by the National University of Singapore Institutional Animal Care and Use Committee (IACUC) (Protocol Number: R2021-0034). First, nCVTs were administered to 4–6-week-old female Balb/c mice (InVivos, Singapore, Singapore) intravenously via the tail vein. The blood was collected via cardiac puncture, and the spleen was harvested 24 h after administration of nCVTs. For immune cells’ profiling, the cells in blood and spleen were isolated and stained with primary antibodies for 30 min on ice, followed by washing with staining buffer twice. The cells were then analyzed by flow cytometry using BD LSRFortessa (BD Biosciences, San Jose, CA, USA). For plasma cytokine analysis, cytokine concentrations were measured using Bio-Plex Pro Mouse Cytokine 23-plex Assay (Bio-Rad, Hercules, CA, USA) according to the manufacturer’s instructions.

### 2.10. Influence of Temperature Block on Cellular Uptake

HeLa and CT26 cells were seeded into black 96-well plates (1 × 10^4^/well) and incubated overnight at 37 °C in an atmosphere of 5% CO_2_. Next, respective plates were pre-cooled to 25 °C and 4 °C for 30 min before further incubation for 1 h with fluorescence-normalized samples. After incubation for 1 h, cells were further incubated for 15 min with 0.1 μg/mL Hoechst 33342 dye. Cells were then washed twice with sterile PBS, and fluorescence measurements were performed by using a microplate reader at 540/570 nm for Cy3 and 361/497 nm for Hoechst 33342.

### 2.11. Influence of Exposure Time on Cellular Uptake

HeLa and CT26 cells were seeded into black 96-well plates (1 × 10^4^/well) and incubated overnight at 37 °C in an atmosphere of 5% CO_2_. Fluorescence-normalized samples were added at the 24, 4, and 1 h time points, respectively, after which cells were further incubated for 15 min with 0.1 μg/mL Hoechst 33342 dye. Cells were then washed twice with sterile PBS, and fluorescence measurements were performed by using a microplate reader at 540/570 nm for Cy3 and 361/497 nm for Hoechst 33342.

### 2.12. Endocytic Inhibitor Assay

HeLa and CT26 cells were seeded into black 96-well plates (1 × 10^4^/well) and incubated overnight at 37 °C in an atmosphere of 5% CO_2_. Next, cells were pretreated with inhibitors at respective concentrations, as shown in Supplementary Table S1 for 30 min. After 30 min of incubation, cells were washed with sterile PBS, and the medium was replaced with fresh medium. Fluorescence-normalized samples were added, and cells were incubated for another 1 h, after which they were further incubated for 15 min with 0.1 μg/mL Hoechst 33342 dye. Cells were then washed twice with sterile PBS, and fluorescence measurements were performed by using a Synergy^TM^ H1 microplate reader at 540/570 nm for Cy3 and 361/497 nm for Hoechst 33342.

### 2.13. Statistical Analysis

All data are reported as the mean ± the standard deviation (SD), unless otherwise stated; one-way analysis of variance (ANOVA) was employed for statistical analysis of the data, followed by Bonferroni *post hoc* tests, using GraphPad Prism software (Version 5). Differences were considered significant at *p*-values < 0.05.

## 3. Results and Discussion

### 3.1. Cell Ghost (CG) Production and Nanovesicle Characterization

**Figure 1:**
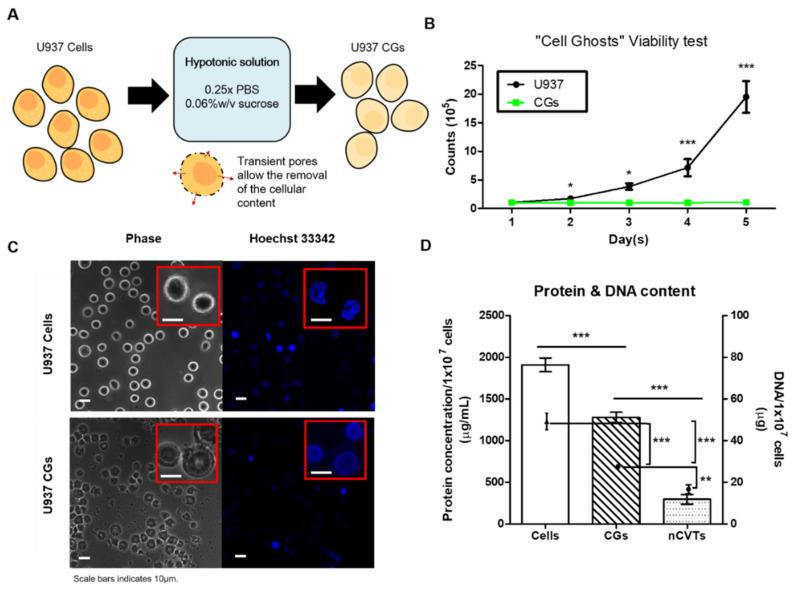
Production of cell ghosts (CGs) from 1 × 10^7^ U937 cells. (**A**) Schematic overview of CGs’ production through hypotonic treatment. (**B**) Cell viability tests of U937, and CGs over the course of 5 days, under the culture condition. (**C**) Representative confocal microscopic images of U937 cells and CGs; insert shows the zoomed-in images of U937 or CGs. Scale bar represents 10 μm. (**D**) Comparison of total protein and DNA concentration of cells, CGs, and nCVTs. All samples were normalized to the same starting cell or CG number of 1 × 10^7^; (*n* = 3/group) * *p* < 0.05, ** p < 0.01 and *** *p* < 0.001.

The cell-ghost production protocol was adopted from our previous study [17]. Briefly, U937 CGs were produced by subjecting the cells to the hypotonic solution (0.06% *w*/*v* sucrose in 0.25× PBS) for 24 h of incubation at room temperature. The sucrose as cryoprotectant was added to ensure the structural integrity of the cell membrane and minimize membrane flipping [17], while the hypotonic solution caused the formation of transient pores to allow for the removal of the cellular content, i.e., cytoplasmic and nuclear content [17]. The addition of the protease inhibitors in the solution also helped to prevent the digestion of the cellular membrane proteins by the cytoplasmic proteases (once released into the medium) (Figure 1A). When CGs were compared against U937 cells in cell culture condition (Figure 1B), they were demonstrated to be non-viable and did not increase in number over days; conversely, U937 cells increased in number exponentially. The lack of viability is an important aspect for the use of any biological reagent (cellular components, in this case), as the introduction of any proliferative agent into host systems may lead to the formation of secondary tumors, activation of the immune system, and disruption of host normal physiology [3].

Furthermore, as seen from the confocal microscopy images (Figure 1C), CGs have more diffused nuclear staining as compared to cells, which have very defined and contrasting stains, indicating that our CG production procedure was able to disrupt the nucleus. Since the nucleus is one of the biggest organelles in cells (consisting of an inner nuclear membrane and an outer nuclear membrane), it was postulated to be the most difficult to be removed. However, when examining the DNA content as an indicator of the nucleus removal (all samples were made from 1 × 10^7^ cells/CGs), our cell-emptying process was able to remove up to 50% of nuclear content while maintaining about 70% of the proteins (Figure 1D). Of note, this DNA content further dropped to about 25% upon the fusion of our CGs with the synthetic lipids in the form of nCVTs, as the process of co-extrusion with synthetic lipids pushed intracellular content farther outside the final nCVTs. The removal of the DNA remnants present in the CGs is also another crucial facet for their potential clinical translation. The introduction of residual DNA may result in the risk of insertional mutagenesis [19] and oncogenesis or even viral infections [20], which subsequently may result in tumor formation. Since our procedures did not aim to preserve the integrity of the DNA fragments, we managed to minimize the remnant DNA. Currently, there is a lack of standard procedure to evaluate such risk: the closest regulatory guideline from the World Health Organization (WHO) and FDA is for biotherapeutics proteins prepared by recombinant DNA technology, which suggests the maximum residual DNA should ideally be kept below 10 ng per parenteral dose [21,22]. However, with emerging uses of extracellular vesicle as potential DDSs or therapeutics, which are known to contain a relatively large amount of DNA from host cells (up to 140 ng of DNA/μg of exosome proteins [23]), our nCVTs with about 25 ng of DNA/μg of nCVTs proteins favorably compared against these extracellular vesicles. Additional advantages of nCVTs over extracellular vesicles include narrower polydispersity (hence, better uniformity), excellent drug loading efficiencies (much higher than extracellular vesicles and comparable to liposomes, which are regarded as gold standard in drug delivery), and higher scalability potential. Nevertheless, additional characterizations and new regulatory guidelines will be needed to further validate key attributes and safety profile of these vesicles.

Both liposomes and nCVTs were prepared with the same pre-optimized lipid formulation of DOPC and cholesterol in a 70 to 30 mole percentage ratio, respectively. The characterization of these nanovesicles is summarized in Supplementary Figure S1 and Table S1.

### 3.2. In Vitro Cytotoxicity and Immunostimulatory Potential

To assess the potential cytotoxic effect of nCVTs upon incubation, we conducted a cell cytotoxicity assay on four different cell lines: two cancer cell lines, HeLa and CT26; one non-cancerous cell line, i.e., HEK293 cells; and one immune cell line, i.e., RAW264.7 cells. Liposomes were used as a control for comparison. The two formulations were normalized according to lipid concentrations (1 mg/mL of synthetic lipid content) and incubated with cells for 72 h. Overall, incubation with both nCVTs and liposomes did not result in any significant cytotoxicity toward all the tested cell lines (Supplementary Figure S2). The only exception was for prolonged incubation times (72 h) in RAW264.7 cells, resulting in cell viability following a dose-dependent trend. Of note, for all subsequent in vitro studies, the concentration of nanovesicles used (<100 μg/mL of lipids) did not lead to significant cytotoxicity. Generally, nCVTs displayed cytotoxic profiles toward all cell lines tested similar to liposomes, which are considered biocompatible gold standards in numerous works in the literature [24]. This suggests that the incorporation of cellular components into liposomes to form nCVTs did not negatively impact the biocompatibility of the formulation.

Apart from the cytotoxic effects, we also attempted to investigate the immunostimulatory potential of nCVTs. Macrophages are immune cells that play a central role in both innate and adaptive immunity by participating in a wide variety of biological processes [25]. Furthermore, macrophages are one of the earliest cell types that process nanoparticles and mediate the downstream host inflammatory and immunological responses [26]. Uptake and accumulation of nanoparticles in macrophages within clearance organs (such as liver and spleen) can lead to the initiation of inflammatory responses due to non-specific recognition, inducing subsequent immunological responses and toxicity [27,28]. Thus, we used RAW264.7 macrophages as a model to study if incubation with nCVTs would lead to their activation. Upon exposure to an immune stimulant (such as LPS), macrophages are known to produce nitric oxide (NO); thus, we used the production of NO as a marker for activated macrophages. Furthermore, NO (especially at high concentration) is known to be a pro-inflammatory mediator that can lead to a cascade of inflammatory reactions and even immunogenicity [29]. Hence, the production of NO can serve as a surrogate indicator for the immunogenic potential of the formulation. LPS was selected as a positive control, as it is a known inflammatory mediator [27].

**Figure 2:**
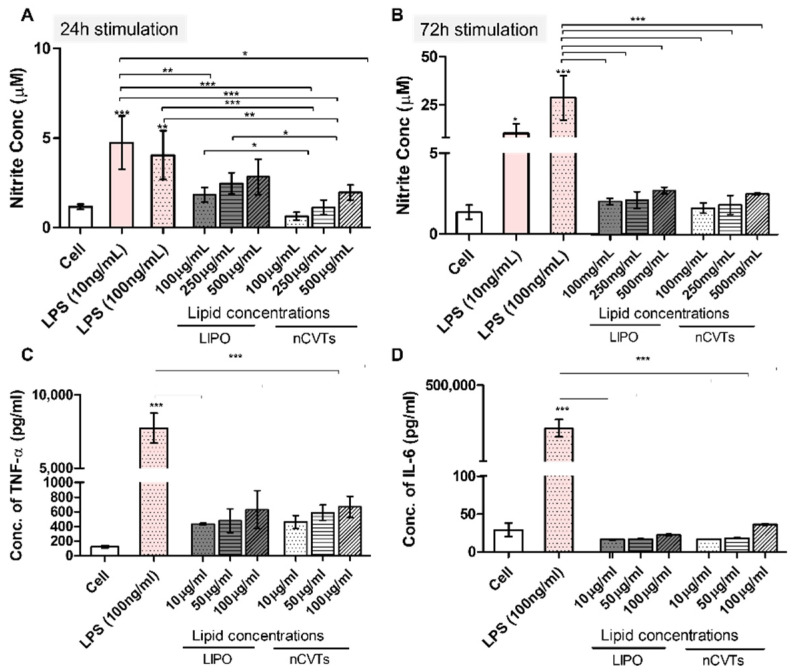
Immunological profile of nCVTs and liposomes (LIPOs). NO production upon treatment of nCVTs and liposomes after (**A**) 24 h of stimulation and (**B**) 72 h of stimulation. Production of pro-inflammatory cytokines: (**C**) TNF-α and (**D**) IL-6 by RAW264.7 cells after 72 h of treatment of respective formulations; (*n* = 3/groups) * p < 0.05, ** p < 0.01, and *** *p* < 0.001.

As seen in Figure 2A, the amount of NO produced by RAW264.7 cells increased with the increasing concentration of nanovesicles after 24 h of treatment, albeit at different rates. Interestingly, when a lower concentration was used, liposomes were found to induce a larger amount of NO production than nCVTs, while this difference was absolved when higher concentrations of nanovesicles were used. This difference between nCVTs and liposomes became less distinguishable upon prolonged incubation (72 h) (Figure 2B). This is indicative that nCVTs have a lower immunostimulatory potential than liposomes (at least at lower concentrations), and this could be ascribed to the retention of various components of the U937 cellular membrane that enhance their biocompatibility and reduce their eventual immunogenicity. Since liposomes lack these components, they could be perceived as “more foreigner” by cells and potentially cause greater activation of macrophages compared to nCVTs [30]. Nonetheless, as the amount of NO induced by both nanovesicles across all concentrations and time points were significantly lower than LPS induction (at both 10 and 100 ng/mL), it could be inferred that our formulation did not significantly induce the activation of macrophages or lead to inflammation.

To further investigate the immunological profile of nCVTs, we measured the secretion of pro-inflammatory cytokines (such as IL-6 and TNF-α) from macrophages upon prolonged treatment (i.e., 72 h) with nanovesicles (Figure 2C,D). Different doses (based on lipid concentrations) were selected according to their cell viability in order to ensure that cytotoxicity did not play a role in inducing the production of cytokines [31]. Particularly, the release of pro-inflammatory cytokines can result in acute immune responses through initiating a positive feedback loop between cytokines and immune cells [32,33]. At a higher nanovesicle concentration, we observed a slight increase in the production of cytokines; however, this slight induction, similar to NO production, was significantly lower than the positive control (i.e., LPS induction).

### 3.3. In Vivo Immunogenicity of nCVTs

**Figure 3:**
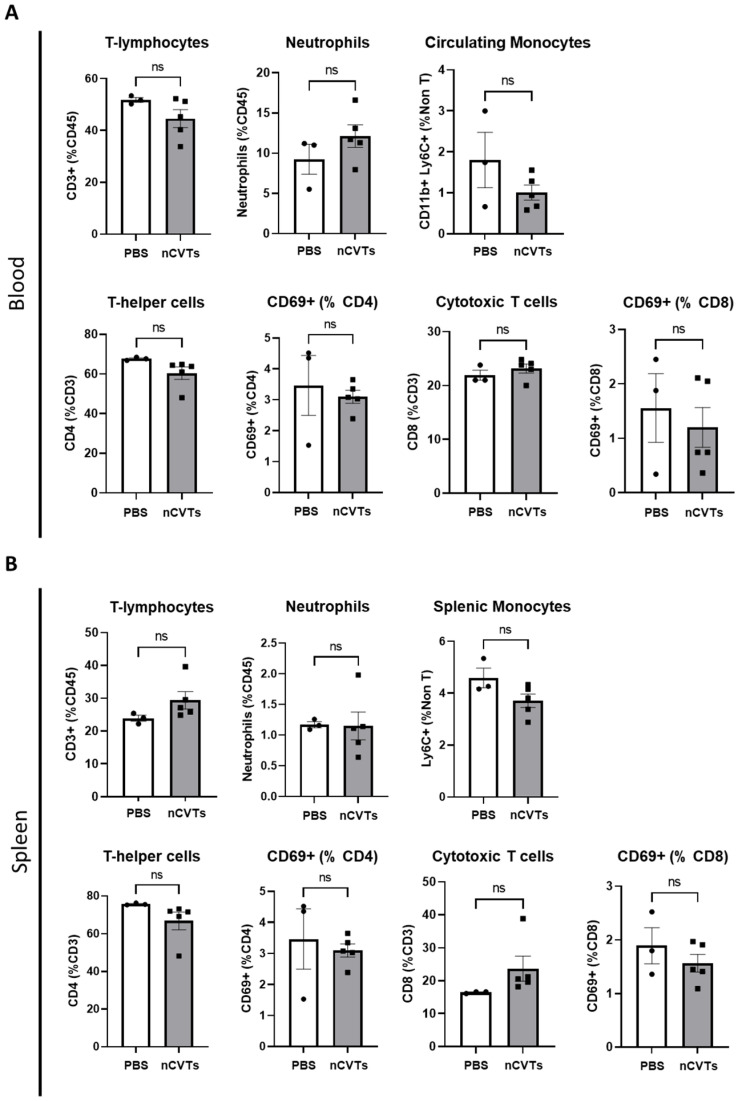
Immune cell profiles in the (**A**) blood and (**B**) spleen (*n* = 3 for PBS group; *n* = 5 for nCVTs group). ns indicates not significant (*p* > 0.05).

To assess the immunogenicity of nCVTs in vivo, 150 µL of nCVTs was injected intravenously in Balb/c mice. Twenty-four hours after the administration of nCVTs, the blood was isolated, and the spleen was harvested. The immune cell profiling of the blood and spleen was analyzed. The immune cell profiling showed no significant differences in the proportion of T-lymphocytes, neutrophils, and monocytes in blood and spleen (Figure 3A,B). There was also no substantial change in the proportion of CD69+ T-lymphocytes, thus signifying no significant activation of T-lymphocytes observed after the administration of nCVTs. This suggests that nCVTs do not alter the immune cell populations in vivo.

**Figure 4:**
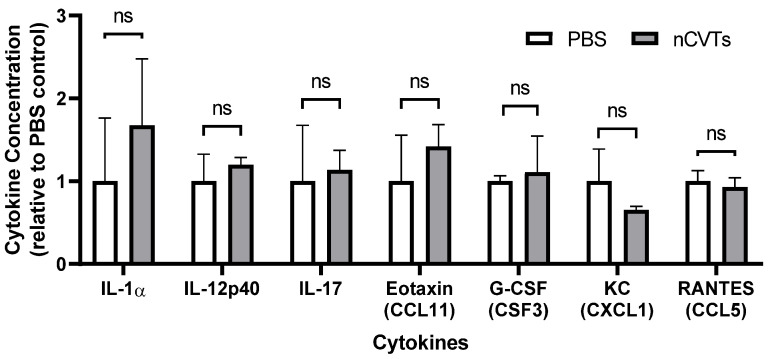
Plasma cytokine concentrations 24 h post-intravenous administration of nCVTs relative to PBS control. Other cytokines (not indicated in the figure) are below the detection limit. ns indicates not significant (*p* > 0.05).

Moreover, we also analyzed the cytokine concentrations in the blood plasma (Figure 4), and the results showed that nCVTs did not have any significant effect on the cytokine concentration in plasma. We then investigated several pro-inflammatory cytokines. IL-1α is a potent inflammatory cytokine that is mainly produced by activated macrophages that trigger the inflammatory process [34]. IL-17 is associated with T-cell-mediated activation of neutrophils and promotes neutrophils’ inflammation [35]. The keratinocytes-derived chemokine (KC) mediates neutrophil recruitment. Moreover, in this case, nCVTs did not stimulate these pro-inflammatory interleukins [36]. There was also no stimulation of pro-inflammatory colony stimulating factors and chemokines. Overall, the administration of nCVTs did not elevate pro-inflammatory cytokines and did not change the distribution of immune cells in vivo. This implies that the nCVTs could be a safe drug delivery system with minimal immunological response.

### 3.4. Mechanisms of Cellular Uptake of nCVTs and Liposomes

To investigate the mechanisms of cellular uptake for nCVTs as a hybrid system, we first explored the effect of temperature on the uptake of nCVTs in comparison to the internalization of purely synthetic nanoparticles, such as liposomes, and purely cell-derived nano-CGs. The same dose of nanovesicles was added to HeLa and CT26 cells, respectively, and a cellular uptake study was carried out at different temperatures (4, 25, and 37 °C). Lowering the incubation temperature reduced the cellular uptake of the nanovesicles, as the reduction in temperature contributed to slower cellular processes, such as metabolism, thus concomitantly reducing the overall intracellular energy [37]. This observation confirms that the uptake of these nanoparticles was predominantly driven by energy-dependent processes such as endocytosis (Supplementary Figure S3). This is aligned with the literature that suggests that liposomes are mainly internalized through endocytosis (i.e., clathrin-mediated endocytosis (CLME) [38]) or, to a certain extent, by direct fusion with cell membrane [39]. Likewise, cells uptake cell-derived systems (i.e., exosomes or other extracellular vesicles (EVs)) through various endocytic pathways, especially via the receptor-mediated processes (i.e., CLME and caveolae-mediated endocytosis (CVME) [40,41].

**Figure 5:**
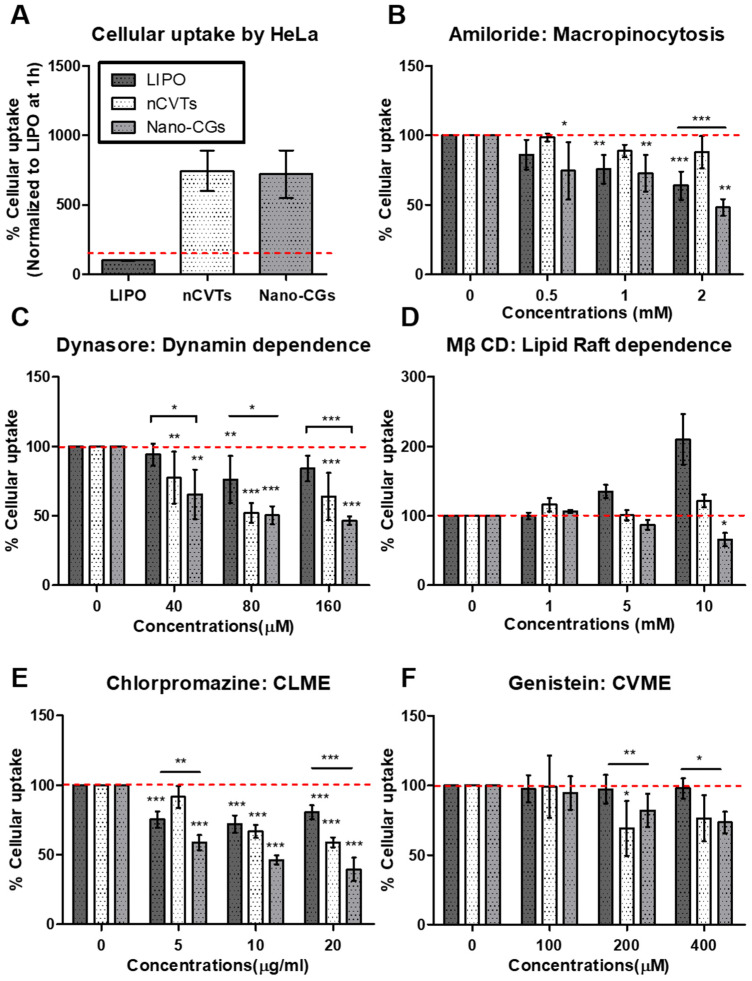
Cellular uptake of liposomes (LIPOs) nCVTs and nano-CGs by HeLa cells (**A**) in absence of inhibitors and the presence of (**B**) amiloride, (**C**) dynasore, (**D**) methyl-β cyclodextrin (MβCD), (**E**) chlorpromazine, and (**F**) genistein; (*n* = 5/group) * p < 0.05, ** p < 0.01, and *** *p* < 0.001.

**Figure 6:**
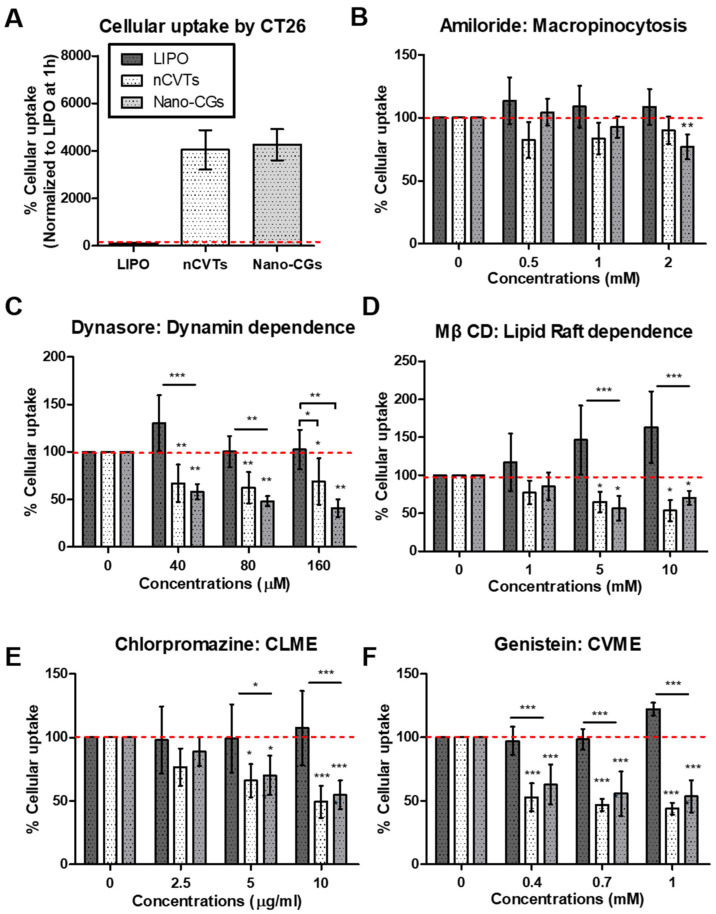
Cellular uptake of liposomes (LIPOs) nCVTs and nan-CGs by CT26 cells (**A**) in the absence of inhibitors and the presence of (**B**) amiloride, (**C**) dynasore, (**D**) methyl-β cyclodextrin (MβCD) (**E**) chlorpromazine, and (**F**) genistein; (*n* = 5/group) * p < 0.05, ** p < 0.01, and *** *p* < 0.001.

To further explore the endocytic mechanism of uptake, different inhibitors were used (Supplementary Table S1). The cells were pretreated with the respective inhibitors to retard a specific mechanism of uptake. Three concentrations of the inhibitors were used to demonstrate a dose-dependent inhibition. The uptake of nCVTs was compared with the synthetic liposomes (with same lipid formulation) and nano-CGs to elucidate the effect of the cellular component in nCVTs’ uptake. Generally, the cellular uptake of nCVTs and nano-CGs was much higher than the uptake in liposomes (Figure 5A and Figure 6A); thus, we normalized the uptake to their respective controls (cellular uptake in absence of inhibitors) to examine the mechanism of uptake for different nanovesicles.

To investigate the role of macropinocytosis in the internalization of nanoparticles, cells were pretreated with amiloride. Amiloride is a known inhibitor of macropinocytosis, and it acts by inhibiting Na^+^/H^+^ exchange, which consequently leads to intracellular acidification [42]. The maximum possible concentration of amiloride was capped at 2 mM, as beyond this value, it would exceed the critical concentration of 1% (*v/v*) of DMSO (amiloride DMSO stock 200 mM) in the cell culture medium. Nonetheless, it was observed that, in HeLa cells (Figure 5B), only the uptake of liposomes and nano-CGs showed dose-dependent inhibitions; meanwhile the uptake of nCVTs was not affected even at the maximum concentration of 2 mM. Conversely, in CT26 cells (Figure 6B), all nanovesicles did not show significant inhibition or any dose-dependent effect; only at a maximum concentration of 2 mM, a slight inhibition was observed for nano-CGs. The decreasing trend of liposomes’ uptake in HeLa is indicative that macropinocytosis is involved in the internalization of liposomes. Since macropinocytosis is a non-selective pathway that internalizes particles of size less than 5 μm, little or no inhibition in the uptake of nCVTs by amiloride may suggest that nCVTs’ uptake involved more selective pathways. Interestingly, nano-CGs also displayed a dose-dependent inhibition by amiloride, indicating that macropinocytosis was involved in the uptake of nano-CGs. Similar results have also been reported for the uptake of EVs in the literature, indicating the involvement of this non-selective pathway in the uptake of cell-derived nanovesicles. Such an observation is likely contributed to by the small size of EVs (<150 nm), as this caused them to be more susceptible to being engulfed together with the extracellular matrix [43,44].

Dynasore, an established inhibitor of GTPase dynamin, was used to inhibit dynamine-dependent pathways [45]. It was observed that the uptake of liposomes was slightly inhibited by dynasore in HeLa cells at 80 μM (about 15%), while their uptake was not significantly inhibited at all concentrations tested in CT26 cells. Comparatively, dynasore inhibited nCVTs’ and nano-CGs’ uptake much more significantly in both HeLa and CT26 cells. In HeLa cells (Figure 5C), the inhibition increased and reached a plateau at 80 μM (about 50%), while inhibition of nCVTs and nano-CGs uptake in CT26 (about 40% for nCVTs and about 60% for nano-CGs at 80μM) did not show a significant dose-dependent effect (Figure 6C). Inhibition of nano-CGs’ and nCVTs’ uptake by dynasore suggests that the internalization of nano-CGs and nCVTs by both HeLa and CT26 was via dynamin-dependent pathways such as CLME and CVME (also possibly some CCIE, which also involved GTPase dynamin). Other cell-derived vesicles (i.e., EVs) have also been reported to take advantage of the dynamin-dependent pathways through interaction with specific receptors [46]. As a hybrid system of cellular components and synthetic lipids, nCVTs may acquire surface membrane cues from parent U937 cells that enable their interaction with receptors involved in receptor-mediated endocytosis (i.e., CLME and CVME), thus leading to a greater dependence on this pathway than purely synthetic liposomes.

In addition, a cell-membrane cholesterol-depleting agent, MβCD, was used to investigate lipid raft-dependent endocytosis. In both HeLa and CT26 cells, the uptake of liposomes was not inhibited by MβCD at any tested concentration; instead, we observed a compensatory uptake mechanism for liposomes when higher concentrations of MβCD were used [47]. However, while in HeLa cells (Figure 5D) we did not observe any significant inhibition of nano-CGs and nCVTs uptake at all concentrations of MβCD tested (except at 10 mM, where a slight inhibition of nano-CGs was observed), the uptake of nano-CGs and nCVTs was significantly inhibited in CT26 (about 40 to 50% with 5 or 10 mM of MβCD), suggesting the involvement of lipid raft-dependent pathways, such as CVME or some CCIE, in the uptake of these nanoparticles by CT26 cells (Figure 6D). Similar to other reports in the literature on cell-derived vesicles (mainly exosomes and other EVs) that showed that lipid rafts were involved in their uptake, the lipid-raft dependence of nano-CGs’ uptake can possibly be attributed to the interaction of receptors and ligands between these cell-derived nanoparticles and those present within the raft domains of the recipient cells [41,48]. On the other hand, a plausible explanation for the involvement of lipid raft-dependent endocytosis in nCVTs’ uptake is that, as a hybrid system, nCVTs retained the cell membrane ligands (such as membrane-bound growth factors [49]) from parent U937 cells, which facilitated the interaction with corresponding receptors (i.e., receptor tyrosine kinases and some transmembrane receptors [49]) on CT26 cells, thus triggering the internalization. Moreover, nCVTs may also inherit the cholesterol-enriched lipid rafts from the U937 cellular components (i.e., CGs), which further enhance their interactions with lipid-rafts found on the CT26 cells via hydrophobic interactions [50]. The lack of these collective features on liposomes may explain the limited involvement of their receptor-mediated uptake.

Chlorpromazine is a cationic amphiphilic molecule that is known to inhibit CLME by blocking clathrin disassembly and its receptor recycling [51]. CLME has been demonstrated to be a major pathway for the uptake of nanoparticles with a size of about 200 nm. Being below 200 nm, all nanoparticles were expected to be internalized through this pathway. Indeed, in HeLa cells, the uptake of nanoparticles was inhibited by chlorpromazine, with nCVTs and nano-CGs showing higher inhibition (43.3% for nCVTs and 53.8% for nano-CGs *versus* 29.1% for liposomes with 10 μg/mL of chlorpromazine; see Figure 5E). This indicates that CLME was involved in the uptake of all tested nanoparticles in HeLa cells. Nonetheless, as a receptor-mediated process, specific recognition of receptors found within the clathrin-coated pits on the cell membrane was required. The ability of cell-derived vesicles (i.e., EVs) to activate specific receptors that triggered CLME has been reported extensively in the literature [43,52]. Considering that the surface of the nCVTs is decorated with membrane proteins and ligands (i.e., with various glycans, membrane-bound growth factors, and chemokines/interleukins [49]) of U937 cells, these ligands may be capable of activating the receptors (such as G-protein-coupled receptors, receptors tyrosine kinase, and other transmembrane receptors [49]) and facilitate nCVTs’ uptake via CLME. Comparatively, liposomes do not have any protein-decorated surface; thus, they have limited ability to activate this receptor-mediated uptake. The observation in CT26 cells further illustrates this point (Figure 6E), as only the uptake of nCVTs (but not liposomes), was significantly inhibited by chlorpromazine at all the concentrations tested. As a dynamin-dependent pathway, this set of data also corroborates the profiles observed with the treatment of dynasore (an inhibitor of dynamin-dependent pathway), as described earlier.

CVME was inhibited by using genistein, an inhibitor that blocks the activities of tyrosine kinases involved in the caveolae pinching [53]. In both HeLa and CT26 cells, only the uptake of nCVTs and nano-CGs was significantly inhibited, suggesting the involvement of CVME in the uptake of nCVTs and nano-CGs in both cell lines. Nano-CGs (120 nm), similar to EVs, as reported in other studies, can be internalized by cells via CVME [54]. Interestingly, though CVME was typically reported to be involved in the internalization of particles with a size range of 50 to 80 nm [55], nCVTs with a size of around 200 nm were able to be at least partially internalized via this pathway. Plausibly, due to the fact that nCVTs have a PDI of about 0.2, they consisted of a heterogeneous system, whereby some vesicles with smaller sizes (in the range of 50 to 80 nm) were affected by this inhibitory process. Alternatively, as nCVTs consisted of ligands and surface membrane cues which enable nCVTs to stimulate clustering and invagination of cell membrane [56], they might have promoted internalization of nCVTs via CVME. As a dynamin- and lipid-raft-dependent pathway, the uptake profile obtained from cells treated with genistein was in agreement with the profiles from cells treated with dynasore and MβCD, respectively. Of note, the uptake of nCVTs through the CVME pathway may help to protect nCVTs from endolysosomal degradation, as it has been reported that nanoparticles’ uptake through CVME can bypass the lysosomal degradation through a distinct cellular compartment [57,58]. The ability to prevent degradation from the endolysosomal system may facilitate nCVTs to efficiently deliver bioactive molecules.

**Figure 7:**
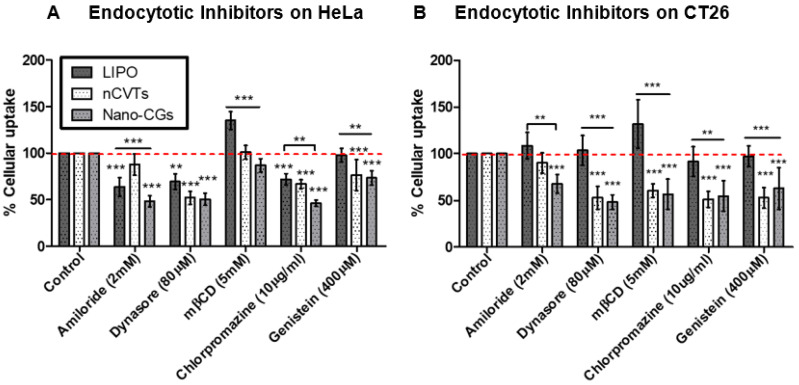
Summary of uptake of nCVTs, liposomes (LIPOs), and nano-CGs in the presence of respective inhibitors by (**A**) HeLa cells and (**B**) CT26 cells; (*n* = 5/group) ** p < 0.01, and *** *p* < 0.001.

Overall, from Figure 7A, micropinocytosis only plays a role in the uptake of liposomes but not nCVTs in HeLa cells. Uptake of liposomes by HeLa cells was also found to be partially contributed by CLME. In comparison, nCVTs were internalized by HeLa and CT26 cells through CLME and CVME, while none of the inhibitors of these pathways showed a significant reduction in uptake of liposomes by CT26 cells (Figure 7B). This suggests a possible involvement of other uptake mechanisms for liposomes, such as direct fusion or perhaps CCIE pathways that did not depend on dynamin or lipid raft [36].

It is noteworthy that nCVTs’ uptake, similar to nano-CGs, was dominated by receptor-mediated processes (i.e., CLME and CVME), and thus evidently suggests that nCVTs inherited surface cues from parent U937 cells, and these features can further enhance the receptor interaction to trigger uptake via respective receptor-mediated pathways. Notably, macropinocytosis was involved in the uptake of nano-CGs but not nCVTs, probably due to the fact that nano-CGs (about 120 nm) were smaller than nCVTs (about 200 nm), and thus, they indirectly became more susceptible to being internalized when cells non-specifically engulf the extracellular materials via macropinocytosis. Nonetheless, considering nCVTs as a complex system produced from the fusion of synthetic lipids and cellular components, having a heterogeneous population of vesicles in a broader range of sizes, it is likely that not a single mechanism but, rather, an interplay of several phenomena contributed to the uptake of nCVTs.

## 4. Conclusions

As a hybrid system of cellular components and synthetic lipids, nCVTs represent an intriguing DDS for cancer therapy. We developed a “cell emptying” procedure to remove the unnecessary cellular contents, while preserving the cell membrane structures to ensure the safety profile of the vesicles. In addition, nCVTs displayed a comparable cytotoxic and immunological profile to conventional liposomal formulations, suggesting their good biocompatibility and low toxicity. We also showed that nCVTs did not produce any significant immunogenic effects when administered in vivo. In addition, we investigated the major endocytic pathways for internalization of nCVTs. In view of ligands/proteins inherited from parent U937 cells, nCVTs were shown to be internalized by cancer cells mainly through receptor-mediated endocytosis, further highlighting the involvement of specific interactions (i.e., receptor–ligand interactions) in uptake of nCVTs. Future studies can further exploit the uptake of nCVTs through the rational design of surface components on these bio-hybrids: this can be achieved by either bioengineering (e.g., genetic modification of parent cells to increase the expression of a certain ligand on the cellular membrane [59,60], followed by nCVT production) or chemical engineering (e.g., strain-promoted alkyne-azide cycloaddition (SPAAC) strategies [61,62,63]. One possibility is, for example, to include LFA-1 or αLβ2 integrin on the surface of nCVTs, as both have been shown to interact with multiple cell-adhesion molecules that become overexpressed at the diseased area (e.g., tumor). This would confer more selective cellular uptake of nCVTs at the tumor sites, thus paving the way for the development of next-generation targeted DDS.

## Data Availability

Data is contained within the article or supplementary material.

## Supplementary Materials

The following supporting information can be downloaded at https://www.mdpi.com/article/10.3390/pharmaceutics14081738/s1. Figure S1: (top) Size distribution of liposomes and nCVTs by density. (bottom) The stability of DOPC:Cho (70:30 mol%) formulated nCVTs and Liposomes (LIPO) over a time course of 3 months under that storage condition of 4°C. No significant changes were observed in size and PDI through for both liposomes and nCVTs, indicating a good stability (n = 3/group). Figure S2A: Cytotoxic effect on immune cells after incubation with the respective amount of nCVTs and Liposomes (LIPO) for (A) 24 h and (B) 72 h (n = 3/group). Figure S2B: Effect of Liposomes (LIPO) and nCVTs on cell viability (72 h). (A) HeLa cells, (B) CT26 cells and (C) HEK293 cells (n = 5/group). Figure S3: Effect of temperature on cellular uptake of different nanoparticles in (A) HeLa and (B) CT26. (n = 3/group) * *p* < 0.05, ** *p* < 0.01, *** *p* < 0.001. Table S1. Endocytosis Inhibitors and Respective Concentrations. Clathrin-mediated endocytosis (CLME), Caveolae-mediated endocytosis (CVME), and Clathrin-/Caveolae-Independent Endocytosis (CCIE)).
