# Supplementary material for: Investigations on Cellular Uptake Mechanisms and Immunogenicity Profile of Novel Bio-Hybrid Nanovesicles
Source: Pharmaceutics. 2022 Aug 20;14(8):1738. doi: 10.3390/pharmaceutics14081738 (PMC9413569; doi:10.3390/pharmaceutics14081738)
Supplement: Supplementary file 1 [file pharmaceutics-14-01738-s001.zip › pharmaceutics-1806222-supplementary.pdf]

Supplementary Materials: Investigations on Cellular Uptake Mechanisms and Immunogenicity Profile of Novel Bio-Hybrid Nanovesicles

Yi-Hsuan Ou, Jeremy Liang, Wei Heng Chng, Ram Pravin Kumar Muthuramalingam, Zi Xiu Ng, Choon Keong Lee, Yub Raj Neupane, Jia Ning Nicolette Yau, Sitong Zhang, Charles Kang Liang Lou, Chenyuan Huang [6,8], Jiong-Wei Wang and Giorgia Pastorin

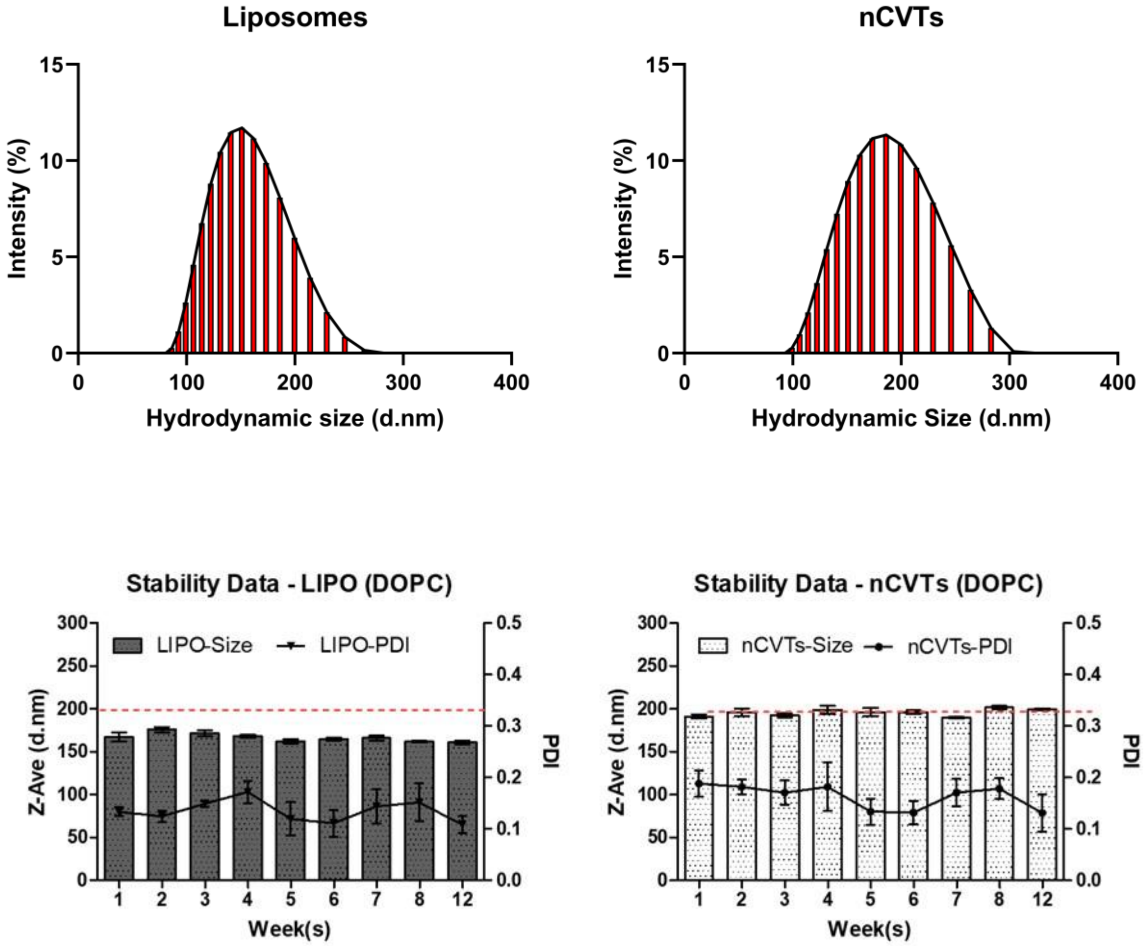

Figure S1: (top) Size distribution of liposomes and nCVTs by density. **(bottom)** The stability of DOPC:Cho (70:30 mol%) formulated nCVTs and Liposomes (LIPO) over a time course of 3 months under that storage condition of 4°C.No significant changes in size and PDI through the course for both liposomes and nCVTs, indicating a good stability (n = 3/group).

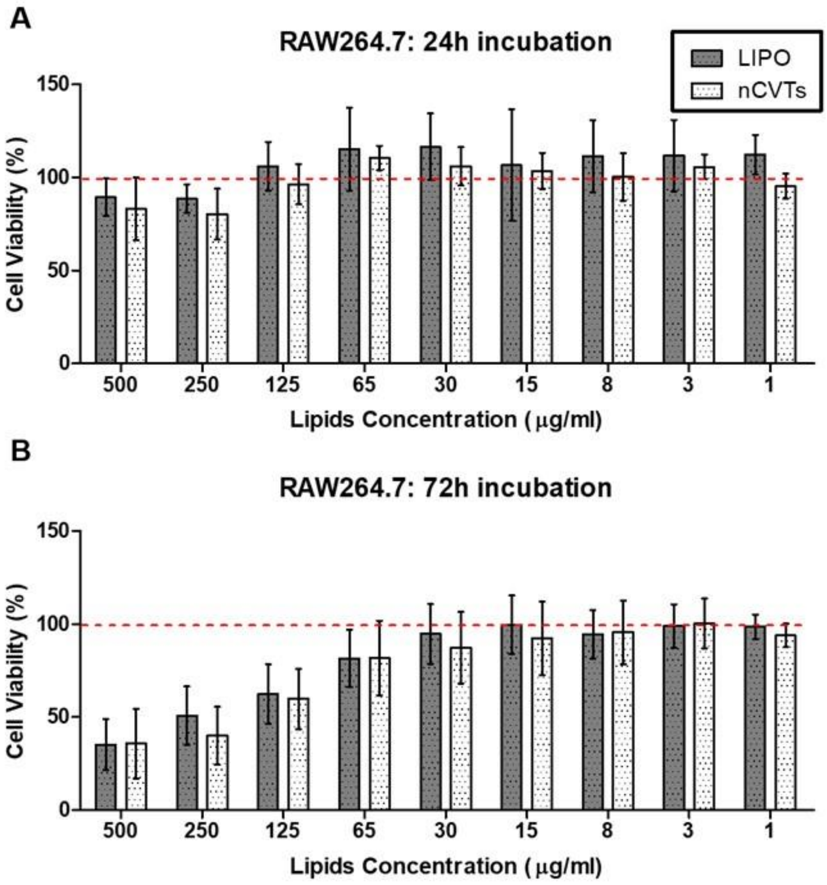

Figure S2A: Cytotoxic effect on immune cells after incubation with the respective amount of nCVTs and Liposomes (LIPO) for (**A**) 24 hours and (**B**) 72 hours (n = 3/group).

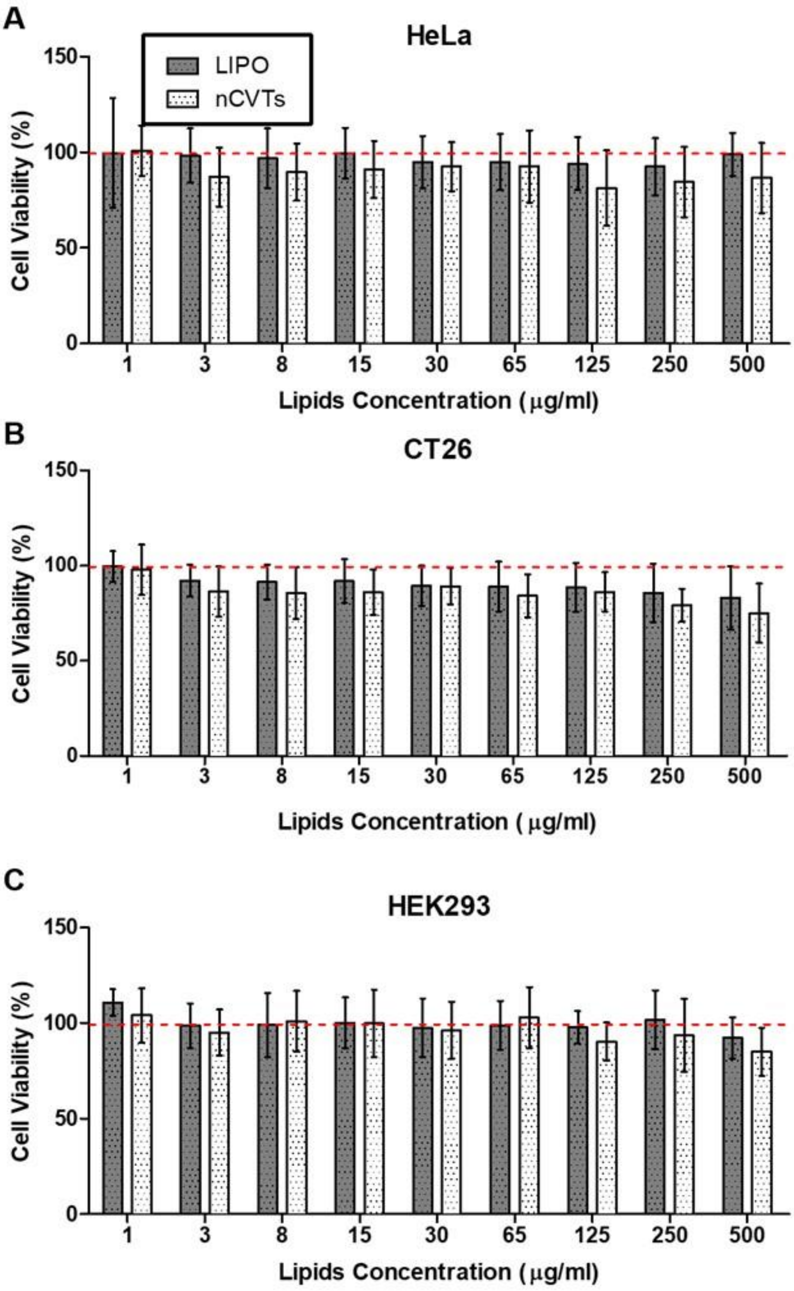

Figure S2B: Effect of Liposomes (LIPO) and nCVTs on cell viability (72 hours). (**A**) HeLa cells, (**B**) CT26 cells and (**C**) HEK293 cells (n = 5/group).

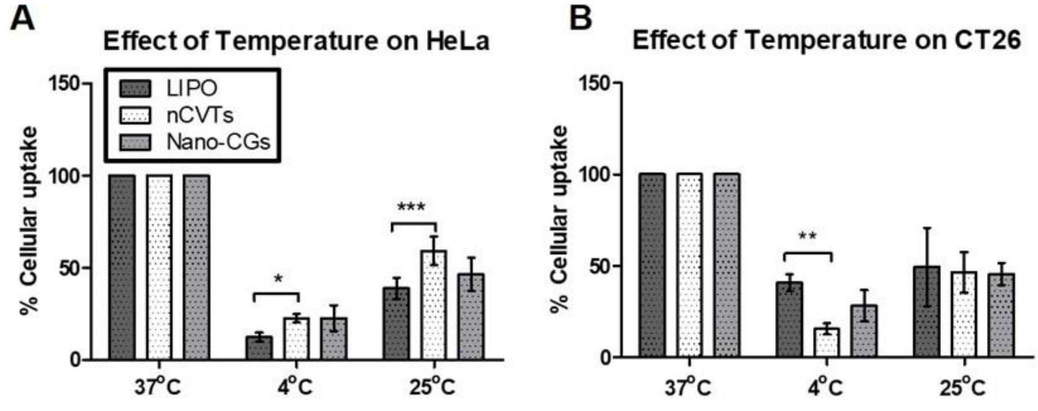

Figure S3: Effect of temperature on cellular uptake of different nanoparticles in (**A**) HeLa and (**B**) CT26. (n = 3/group) **p<0.05, **p<0.01, ***p<0.001.*

Table S1. Endocytotic Inhibitors and Respective Concentrations. Clathrin-mediated endocytosis (CLME), Caveolae-mediated endocytosis (CVME), and Clathrin-/Caveolae-Independent Endocytosis (CCIE)).

**Endocytic Inhibitors**	**Endocytic Pathway Blocked**	**Concentrations of Inhibitors tested**
Amiloride [18]	Macropinocytosis	250 μM, 1 mM, 2 mM
Dynasore [19]	Dynamin-dependent Endocytosis<br>(CLME, CVME, CCIE)	8 μM, 80 μM, 160 μM
Methyl-β Cyclodextrin<br>(MβCD) [19]	Lipid raft-dependent Endocyto-<br>sis<br>(CVME, CCIE)	1 mM, 5 mM, 10 mM
Chlorpromazine [18,19]	CLME	1 μg/ml, 10 μg/ml, 20 μg/ml (in HeLa)<br>2.5 μg/ml, 5 μg/ml, 10 μg/ml (in CT26)
Genistein [18]	CVME	100 μM, 200 μM, 400 μM (in HeLa)<br>400 μM, 700 μM, 1000 μM (in CT26)

CCIE: Clathrin- & Caveolae- Independent Endocytosis.
CLME: Clathrin-mediated Endocytosis
CVME: Caveolae-mediated Endocytosis
